# Multivariate Markovian modeling of tuberculosis: forecast for the United States.

**DOI:** 10.3201/eid0602.000207

**Published:** 2000

**Authors:** S M Debanne, R A Bielefeld, G M Cauthen, T M Daniel, D Y Rowland

**Affiliations:** Department of Epidemiology and Biostatistics, School of Medicine, Case Western Reserve University, Cleveland, OH 44106-4945, USA. smd@hal.cwru.edu

## Abstract

We have developed a computer-implemented, multivariate Markov chain model to project tuberculosis (TB) incidence in the United States from 1980 to 2010 in disaggregated demographic groups. Uncertainty in model parameters and in the projections is represented by fuzzy numbers. Projections are made under the assumption that current TB control measures will remain unchanged for the projection period. The projections of the model demonstrate an intermediate increase in national TB incidence (similar to that which actually occurred) followed by continuing decline. The rate of decline depends strongly on geographic, racial, and ethnic characteristics. The model predicts that the rate of decline in the number of cases among Hispanics will be slower than among white non-Hispanics and black non-Hispanics a prediction supported by the most recent data.


### Multivariate Markovian Modeling of Tuberculosis: Forecast for the United States

**Sara M. Debanne,* Roger A. Bielefeld,* George M. Cauthen,† Thomas M. Daniel,* and Douglas Y. Rowland‡**
*Case Western Reserve University, Cleveland, Ohio, USA;
†Centers for Disease Control and Prevention, Atlanta, Georgia, USA; and
‡D.Y. Rowland Associates, Cleveland, Ohio, USA

We have developed a computer-implemented, multivariate Markov chain model to project tuberculosis (TB) incidence in the United States from 1980 to 2010 in disaggregated demographic groups. Uncertainty in model parameters and in the projections is represented by fuzzy numbers. Projections are made under the assumption that current TB control measures will remain unchanged for the projection period. The projections of the model demonstrate an intermediate increase in national TB incidence (similar to that which actually occurred) followed by continuing decline. The rate of decline depends strongly on geographic, racial, and ethnic characteristics. The model predicts that the rate of decline in the number of cases among Hispanics will be slower than among white non-Hispanics and black non-Hispanics—a prediction supported by the most recent data.

After many years of decline, tuberculosis (TB) cases began to resurge in the United States in 1984, and continued to climb for a decade before declining again (1-4). Reemergent TB did not affect all segments of the population equally. While incidence continued to decline in some geographic areas, it increased in others, notably in the greater New York City area, southern Florida, the border areas of Texas, and urban California. Disadvantaged populations were disproportionately affected, with peak incidences in blacks and Hispanics now seen in young adults. TB in foreign-born persons accounts for nearly 40% of the total annual cases in the United States (5). Finally, HIV infection has played a large part in the recent increase in TB incidence. Effective TB control requires reliable estimates of future secular trends. We have constructed a population dynamics, mechanistic Markov chain model for the epidemiology of TB in the United States by separate modeling for disaggregated groups defined by race, ethnicity, age, and geography. With this model, we have projected TB incidence for the U.S. population at large, as well as for racial and ethnic and geographically defined groups within the population.

Address for correspondence: Sara M. Debanne, Department of Epidemiology and Biostatistics, School of Medicine, Case Western Reserve University, 10900 Euclid Avenue, Cleveland, OH 44106-4945, USA; fax: 216-368-3970; e-mail: smd@hal.cwru.edu.

### The Study

In support of this work, we obtained datasets from the U.S. Bureau of the Census and the Centers for Disease Control and Prevention (6-19).

The epidemiology of TB in the United States has an underlying Markovian nature (20-22). Except in unknown or difficult-to-predict influxes of immigrants, future infections and cases of TB may be probabilistically estimated by knowing sufficiently well the present situation of susceptible, infected, and ill persons; rates of transition from healthy to infected and infected to ill; patterns of contact; and rates of fertility and death. Indeed, a Markov chain is completely specified by an initial state and transition probabilities. To model the future course of TB in the United States, we used the structure depicted in Figure 1.

The U.S. population was disaggregated into states multivariately defined by sociodemographic, health, and disease descriptors: age (single-year

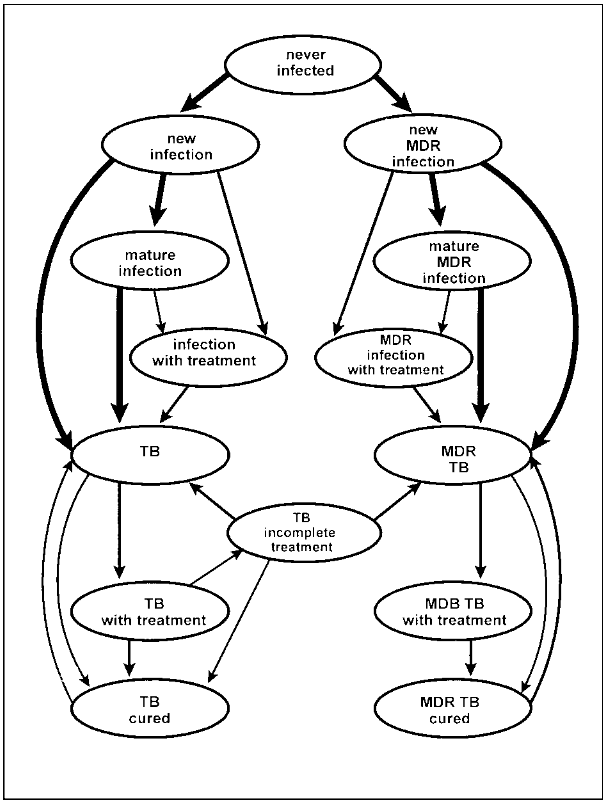

Figure 1. Tuberculosis model states and possible transitions. Thicker lines depict transitions considered in sensitivity analysis.

age groups); sex; race (white, black, Asian or Pacific islander, Native American or Alaskan native, or other race); ethnicity (Hispanic or non-Hispanic); TB infection or disease status; and location. One hundred sixty locations were defined: 60 corresponded to the largest standard metropolitan statistical areas (SMSAs) in the United States, 50 to groupings of smaller SMSAs in the 50 states, and 50 to groupings of non-SMSA counties in the 50 states. The impact of the HIV epidemic on the TB epidemic was addressed by dealing with certain location-specific populations in a way that recognizes that a fraction of them are dually affected. A state in the Markov chain was completely defined by the joint specification of all these descriptors.

In the absence of longitudinal data, the population database for the model was constructed by assembling population profiles for each of the individual years from historical surveys and population projections (5-17,23). These profiles consisted of the sizes of population groups defined multivariately by age, race, sex, ethnicity, and geographic location. The population projections of the Bureau of the Census incorporate expected immigration patterns.

Transition probabilities for the model were based on rates cited in the literature to describe how these transitions occur for individuals. The probability of transition from healthy to infected has been termed the "annual risk for infection." To estimate this probability from tuberculin skin test surveys, we used the catalytic method of Muench (19,24,25). This method relates incidence to prevalence, given several data points of the latter, under the assumption of an exponential model, the exponential curve being called "catalytic" at the time of Muench, who used graphic methods for estimation. The average number of susceptible persons infected by each infectious (i.e., sputum-positive) case in a geographic location in 1 year was termed the "contagious parameter" by Styblo, who noted the correspondence between the prevalence rate of the disease and the annual risk for infection (25,26). Since we assume that new active cases are treated during their year of occurrence, our use of the contagious parameter is consistent with that of Styblo.

An important assumption made in the model about the spread of infection is that each ill person infects susceptible persons entirely within race ethnicity location-specific subgroups, with the number of infections determined by the contagious parameter. The age-sex distribution of newly infected persons in the model was based on national TB contact investigation data (27). Time trends for contagious parameters for the various race ethnicity location-specific subgroups were based on quadratic regression fits of the total cases of TB for those subgroups over time from 1980 through 1993. For illustration, we present in Table 1 baseline values for the contagious parameter for 17- to 25-year-old white men, by state, derived from the U.S. naval recruits data (28-35).

The probability of transition from infected to ill is a product of host-parasite interactions and is determined by the biologic features of these interactions. A person with a new *Mycobacterium tuberculosis* infection (i.e., an infection of <1 year) is at much higher risk for clinical disease than someone with a mature infection, who has been at an ongoing risk in later years (36). These risks vary with age. Although not explicitly modeled, reinfection of cured persons is allowed at the same rate as infections with the same

Table 1. Baseline values of contagious parameters, for 17- to 25-year-old white men, by state

| | New ACR[a] | ARI[b] | ARI/TB[c] |
|---|---|---|---|
| Alabama | 0.13 | 38.2 | 293.6 |
| Arizona | 0.30 | 52.7 | 178.3 |
| Arkansas | 0.15 | 39.7 | 261.6 |
| California | 0.16 | 36.4 | 228.1 |
| Colorado | 0.19 | 18.9 | 97.4 |
| Connecticut | 0.12 | 15.7 | 134.2 |
| Delaware | 0.12 | 30.1 | 243.2 |
| District of Columbia | 0.15 | 55.6 | 376.1 |
| Florida | 0.07 | 27.4 | 410.5 |
| Georgia | 0.08 | 31.8 | 395.7 |
| Idaho | 0.10 | 13.2 | 135.3 |
| Illinois | 0.15 | 39.6 | 269.3 |
| Indiana | 0.16 | 30.7 | 198.0 |
| Iowa | 0.07 | 9.2 | 130.4 |
| Kansas | 0.07 | 12.6 | 176.9 |
| Kentucky | 0.28 | 50.6 | 177.9 |
| Louisiana | 0.10 | 35.4 | 339.6 |
| Maine | 0.14 | 25.2 | 179.9 |
| Maryland | 0.22 | 36.4 | 164.4 |
| Massachusetts | 0.14 | 28.9 | 203.9 |
| Michigan | 0.14 | 29.7 | 205.3 |
| Minnesota | 0.10 | 17.3 | 181.8 |
| Mississippi | 0.09 | 30.3 | 334.3 |
| Missouri | 0.14 | 32.9 | 231.8 |
| Montana | 0.11 | 13.6 | 123.2 |
| Nebraska | 0.09 | 16.4 | 174.5 |
| Nevada | 0.07 | 53.8 | 747.9 |
| New Hampshire | 0.11 | 16.8 | 153.6 |
| New Jersey | 0.15 | 23.7 | 161.6 |
| New Mexico | 0.22 | 29.1 | 130.9 |
| New York | 0.17 | 36.6 | 218.1 |
| North Carolina | 0.08 | 26.1 | 342.8 |
| North Dakota | 0.07 | 8.6 | 122.5 |
| Ohio | 0.14 | 27.1 | 198.2 |
| Oklahoma | 0.13 | 24.0 | 185.8 |
| Oregon | 0.12 | 32.0 | 257.0 |
| Pennsylvania | 0.17 | 32.1 | 183.8 |
| Rhode Island | 0.12 | 27.9 | 235.7 |
| South Carolina | 0.04 | 26.7 | 615.2 |
| South Dakota | 0.06 | 9.2 | 163.8 |
| Tennessee | 0.19 | 45.8 | 244.7 |
| Texas | 0.21 | 29.2 | 138.7 |
| Utah | 0.04 | 7.6 | 197.1 |
| Vermont | 0.18 | 18.3 | 99.3 |
| Virginia | 0.13 | 32.1 | 237.9 |
| Washington | 0.11 | 25.7 | 231.9 |
| West Virginia | 0.21 | 39.1 | 189.4 |
| Wisconsin | 0.08 | 22.1 | 260.1 |
| Wyoming | 0.15 | 10.3 | 66.7 |
| Total | 0.14 | 30.1 | 210.3 |

[a]ACR, Active case rate per 100,000 per year tuberculosis
[b]ARI, Annual rate of infection per 100,000 per year
[c]Contagious parameter characteristics in the population. The case rates among persons with new and mature infections in the next year are based on the upper and lower bounds of rates reported in earlier studies, and are adjusted to calibrate the model to the historical time series (27,37-42) (Table 2).

The effects of the HIV epidemic and the emergence of multidrug-resistant TB are modeled through time-dependent exogenous inputs for appropriate geographic locations and, within these locations, for specific demographic subgroups, as dictated by trends in prevalence and dual-infection rates (43-45). These calculations result in time-dependent changes to the probabilities of transition from new or mature infection to clinical TB.

We established 1980 as the baseline year for all variables used in the model. The initial values of these variables were based on the Reports of Verified Cases of Tuberculosis (RVCT) files and published reports of TB incidence from Centers for Disease Control and Prevention. Because the RVCT program was not implemented nationwide until 1985, reporting for the years 1980 to 1984 was incomplete. To estimate the 1980 baseline values, we inferred values of missing data according to the multivariate structure observed in 1985. Baseline information also included counts of mature infections prevalent in 1980 and counts of new infections in 1980. These values were estimated by applying difference equations relating the numbers of cases, new infections, and mature infections in a given year to the numbers new infections, and mature infections in the previous year, where the relationships between the figures for the 2 years were dependent on model parameters such as survival rates, rates of transition from mature infection to disease, rates of transition from new infection to disease, and contagious parameters, and where each difference equation pertained to a specific multivariately defined subpopulation. Through repeated application of the difference equations, it was possible to express the number of mature infections in 1980 in terms of TB case counts for an arbitrary number of subsequent years for which actual data were available. Estimates thus obtained were examined for consistency and the most stable was selected. Because of the lack of reliable information, we did not attempt to address the underreporting of TB cases or underestimates of some populations.

Table 2. Literature-based lower, best, and upper estimates of the risk of developing tuberculosis for infected persons, by age and sex

| | Acute risk | | Ongoing risk | |
|---|---|---|---|---|
| Age | Male | Female | Male | Female |
| <1 | 0.013, 0.07, 0.07 | 0.013, 0.07, 0.07 | 0.0016, 0.004, 0.009 | 0.0016, 0.004, 0.009 |
| 1-4 | 0.013, 0.026, 0.026 | 0.013, 0.026, 0.026 | 0.0016, 0.004, 0.009 | 0.0016, 0.004, 0.009 |
| 5-14 | 0.002, 0.002, 0.006 | 0.002, 0.002, 0.006 | 0.0008, 0. 0008, 0.0013 | 0.0008, 0.0008, 0.0013 |
| 15-34 | 0.002, 0.01, 0.01 | 0.002, 0.02, 0.02 | 0.0003, 0.0013, 0.0042 | 0.0003, 0.0016, 0.005 |
| 35-54 | 0.0014, 0.009, 0.009 | 0.0014, 0.009, 0.009 | 0.0002, 0.0009, 0.0015 | 0.0002, 0.0009, 0.0015 |
| 55+ | 0.0019, 0.005, 0.005 | 0.0019, 0.005, 0.005 | 0.001, 0.001, 0.002 | 0.001, 0.001, 0.002 |

Computer implementation of the model was carried out on a Sun SPARCstation 5 with 80 megabytes of main memory and network access to a SPARCserver 1000E with approximately 100 gigabytes of online disk storage. All software was written in C++, an object-oriented programming language. A library of C++ objects and functions (46,47) was used as the basis for the representation of fuzzy numbers in the software.

Because of the extremely large sizes of the multidimensional arrays containing cross-sectional population data and year-to-year transition data, specialized data structures and algorithms were developed to take advantage of characteristics of these data that allowed them to be stored in a more compact form (48). For cross-sectional population data, a height-balanced binary search tree structure (49,50) was used. Within each tree node, both cell information and array subscript information were stored. Because the subscripts would occupy at least 9 bytes within each node of the tree if stored conventionally, a 4-byte compressed representation of the subscripts was stored within each node. For transition data, a simple linked-list structure was used in which each node contained both cell information and compressed subscript information. This approach resulted in a 96% reduction of storage space required for population data and a 99% reduction of storage space required for transition data (48).

### Results

Figure 2 presents model projections of new cases of TB for the United States for 1980 through 2010 as well as actual data for white non-Hispanics, blacks, and Hispanics, the groupings used in TB case reporting in the United States. For these three groups, differences between actual counts and model predictions were generally <1,000 for blacks and Hispanics and <1,500 for white non-Hispanics.

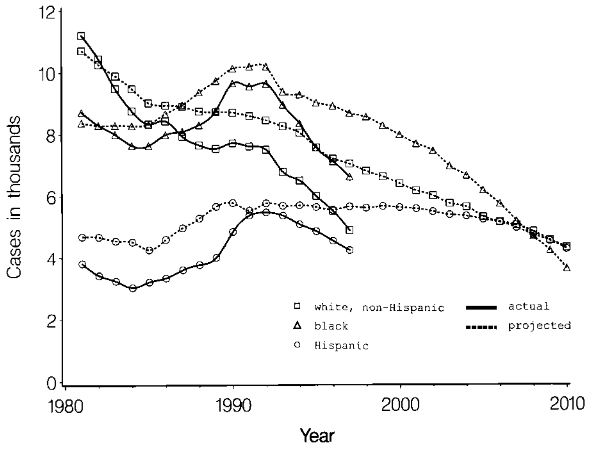

Figure 2. Total number of new cases of tuberculosis in white non-Hispanics, blacks, and Hispanics in the United States, 1980–2010.

Table 3 presents actual and projected TB case rates per 100,000 for each 5 years from 1980 to 2010 with results presented for racial, ethnic, and geographic groups. Generally, the model performs best, within the 1980 to 1995 validation period, for large subgroups with relatively high case rates. For example, it projects TB case rates to within 12% of actual rates for the U.S. population at large. Its performance is not as good in subgroups having lower case rates (e.g., there is a 23% projection error for non-Hispanic whites in 1995). In addition, projection errors are relatively high for smaller subgroups, especially those in which case reporting and population reporting are subject to inaccuracy (e.g., projection errors are consistently above 20% for Hispanics and nearly as high for American Indians and Asian/Pacific Islanders). The model's best performance tends to be among the older population: the model predicts case rates to

Table 3. Actual and projected tuberculosis rates per 100,000 for selected population groups, 1980 to 2010

| | Year | | | | | | | | | |
|---|---|---|---|---|---|---|---|---|---|---|
| | 1980 | 1985 | | 1990 | | 1995 | | 2000 | 2005 | 2010 |
| | Actual | Proj. | Actual | Proj. | Actual | Proj. | Actual | Proj. | Proj. | Proj. |
| All | (12.5) | 10.6 | (9.5) | 11.3 | (10.4) | 9.7 | (8.7) | 8.3 | 6.6 | 4.6 |
| By race/ethnicity | | | | | | | | | | |
| White, not Hispanic | (6.6) | 5.1 | (4.7) | 4.8 | (4.2 ) | 3.8 | (3.1) | 3.2 | 2.6 | 2.1 |
| Black, not Hispanic | (34.3) | 30.5 | (28.0) | 34.7 | (33.0) | 27.9 | (23.9) | 23.1 | 16.9 | 9.3 |
| Hispanic | (25.6) | 24.1 | (18.2) | 26.5 | (22.1) | 33.2 | (18.0) | 28.5 | 23.2 | 16.7 |
| American Indian | (32.7) | 31.5 | (24.5) | 26.3 | (19.5) | 19.2 | (16.5) | 16.6 | 11.4 | 7.0 |
| Asian/Pacific Islander | (86.4) | 39.9 | (50.9) | 34.9 | (42.8) | 27.1 | (45.9) | 20.1 | 13.1 | 6.2 |
| By age | | | | | | | | | | |
| Under 5 | (1.1) | 3.6 | (4.4) | 4.3 | (5.1) | 4.1 | (4.7) | 3.5 | 3.2 | 2.4 |
| 5-14 | (0.8) | 2.5 | (1.4) | 2.4 | (1.9) | 2.2 | (1.7) | 1.9 | 1.7 | 1.3 |
| 15-24 | (2.8) | 6.0 | (4.3) | 8.1 | (5.2) | 6.9 | (4.7) | 5.8 | 4.3 | 3.0 |
| 25-44 | (8.4) | 10.6 | (9.4) | 12.5 | (12.0) | 10.7 | (9.9) | 9.3 | 7.3 | 4.4 |
| 45-64 | (19.6) | 14.0 | (14.0) | 15.3 | (13.7) | 13.6 | (11.5) | 11.7 | 8.9 | 5.9 |
| 65+ | (49.9) | 27.1 | (24.8) | 20.1 | (20.3) | 16.1 | (16.0) | 13.4 | 11.1 | 8.5 |
| Selected Metropolitan Areas | | | | | | | | | | |
| New York City | (18.6) | 24.9 | (24.7) | 41.7 | (43.9) | 35.2 | (30.1) | 30.3 | 25.3 | 17.9 |
| San Francisco | (19.8) | 13.8 | (16.7) | 17.9 | (21.9) | 18.3 | (23.0) | 17.4 | 13.2 | 5.1 |
| Los Angeles | (24.8) | 26.7 | (19.2) | 30.5 | (23.6) | 31.5 | (19.1) | 27.7 | 22.1 | 15.6 |
| Miami | (28.0) | 17.5 | (23.7) | 21.1 | (23.6) | 13.3 | (19.1) | 9.9 | 8.8 | 5.7 |

within 1% accuracy for the U.S. population aged 65 and over for 1990 and 1995. This is of significance in that the highest TB case rates are among the elderly.

Figure 3 presents model projections of the TB case rate quartiles, by state, for the year 2010. Note that the projected case rate for Wyoming spuriously falls into the highest quartile, as a result of its small population size. TB is projected to decrease in all parts of the United States and remain largely a problem of states receiving large numbers of immigrants.

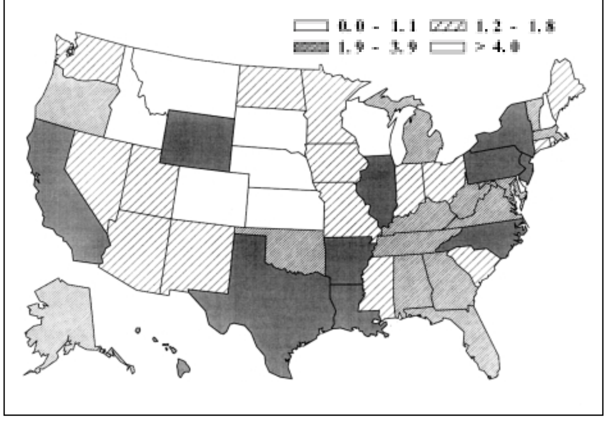

Figure 3. Projected quartiles of tuberculosis case rates per 100,000 by state, 2010.

In Figure 4, the model projection for the United States for 1980 through 2010 is presented together with actual numbers of newly reported TB cases for 1980 through 1997. Also depicted is the sensitivity of the model to changes in key parameters, seen in thicker lines in Figure 1. Specifically, changes were allowed for the contagious parameters and for rates of transition from healthy to infected and infected to ill.

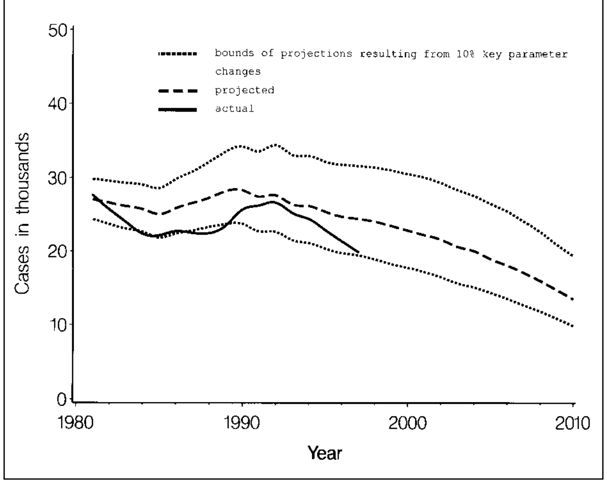

Figure 4. Total number of new cases of tuberculosis in the United States, 1980–2010 and sensitivity of model projections.

Specifically, above and below the central projection are lines representing projections from simultaneous 10% increases and decreases in these key parameters for each year in the projection period. A simultaneous decrease of 10% in the parameters for each year of the projection period results in a decrease of 27% (from 13,458 to 9,861) in projected TB cases in 2010. A simultaneous increase of 10% yields an increase of 44% (from 13,458 to 19,381) in projected cases in 2010. Not shown in the figure, a simultaneous decrease of 5% in the same parameters yields a decrease of 15% (from 13,458 to 11,459) for projected cases in 2010, and a simultaneous increase of 5% yields an increase of 19% (from 13,458 to 16,063) in 2010.

### Conclusions

The studies of Waaler and colleagues, who carefully noted and credited other early workers in this field, mark the beginning of a modern approach to modeling and the thoughtful consideration of transition probabilities in modeling (51,52). ReVelle, Lynn, and Feldman, using many of the assumptions of Waaler and his colleagues, projected declining TB case rates in developing countries as a result of BCG vaccination (53). Azuma modeled TB incidence in Japan (54). Estimates of AIDS prevalence were used by Styblo (55), Schulzer and colleagues (56), and Heymann (57) to project TB incidence in sub-Saharan Africa. Modeling was used by Murray, Styblo, and Rouillon to examine the economic impact of TB in developing countries (58). Blower and colleagues used a differential equation-based model to examine the decline of TB epidemics and emphasize the importance of effective case treatment in TB control (59,60). Murray and colleagues used differential equation-based models to compare the global impact of different control strategies (61,62). Brewer and colleagues used simulation techniques to compare TB control policies (63).

Mathematical modeling of TB in the United States probably began in 1939 with the early work of Frost, who used age cohort analyses of secular trends to predict the future decline of TB (64). Ferebee developed a model for TB in the United States based on data from 1963 and used it to emphasize the potentially important contribution of chemoprophylaxis to TB control (65). The model we present is substantially more complex than Ferebee's and differs from her early work by disaggregating the American population. Given the marked heterogeneity of the population of the United States and the corresponding diversity of TB case rates in various American population groups, our approach offers major advantages over aggregate modeling both in improved accuracy of estimation of input data and in output that can be disaggregated to relate to segments of the population with very different TB control problems.

A highly disaggregated model incorporating population dynamics is appropriate. When applied to small groups, population dynamics are aptly described by a linear model, particularly when natural constraints are stated as totals. The model we constructed is Markovian in that it is independent of its history prior to its last known state. Our computer implementation of the theoretical model is clearly Markovian because its operation parallels the nature of a Markov chain, in the sense that the initial state was the state of our system in 1980, and the state in each subsequent year was generated from the previous year's state and 1-year transition rates. Since no information other than the previous year's state and 1-year transition rates are available to the computer software to generate the state for a year, the Markovian nature is established.

Our model for the intermediate years predicted (on the basis of 1980 baseline data) that TB would increase late in the ensuing decade and decline again. This prediction reasonably approximated the subsequent events. While the model does include changes in TB incidence related to the HIV epidemic and to immigration, it does not specifically incorporate changes in TB control measures, whose impacts are only addressed indirectly through net changes of transition probabilities in the model. The latter could be addressed by a submodel for appropriate population subgroups, and could affect projections.

The projections presented here suggest that the number of new cases and incidence rates of TB will now continue to decrease to a level approximately half the present level nationally. Measuring the extent to which increased control efforts contributed to the return of the TB epidemic to its formerly declining phase will require more detailed analysis.

The handling of geographic location necessarily relies on politically defined entities which determine how data are collected. Since disease patterns do not necessarily follow these boundaries, the model is not uniformly accurate by geographic location. This may be seen in Table 3 where the TB case rate in 1995 is overestimated for Los Angeles and underestimated for Miami. However, the very fine age structure imposed has resulted in projections by age group within 20% accuracy for age 25 and older (Table 3). Refinement of the geographic characterization could result in corresponding increases in accuracy.

The model projections are smoother over time than the actual counts, as should be expected because random fluctuations are not built into the model (Figures 2,3). In Figure 4 the time series of actual cases is moving toward the lower 10% bound. This may be a temporary fluctuation (as in the mid-1980s) but could also indicate changes in transitions in the real-world disease process.

Errors of a projection model may arise from shortcomings of model structure, from inaccuracies in initial conditions, or from errors in the values of key model parameters. The structure of the Markov chain model is mechanistic, driven by highly detailed population dynamics. Each part of the mechanistic process may prompt suggestions for model refinements and discussions of variability; notably, occurrence of infection (66,67), time from infection to disease (68,69), and involvement of HIV (70) and immigrant status (71). The greatest potential for model inaccuracy due to initial conditions arises from estimates of the 1980 pool of prevalent infections. Errors thus introduced become less consequential with passing time, as persons infected before 1980 die. So, model projections in the latter part of the projection period are least affected by errors in the numbers of those infected before 1980. Model parameters not only have the obvious role of governing transitions for various population subgroups, but also, as reflected by their time dependency, are used to depict exogenous phenomena such as the HIV epidemic, implementation of TB control efforts, and changes in immigration patterns. Geographically focused changes of TB control efforts implemented after 1993 related to directly observed therapy could result in overestimation in projections in those areas.

Although the model does not allow mixing of groups from one location to another, this limitation is in large measure adjusted in the calculations of transition probabilities since these are based on actual data that implicitly reflect the movements of healthy, infected, and ill groups. The other type of mixing not accounted for in the model occurs in a location among different race-ethnicity subgroups. The most serious projection errors would then occur when a subgroup of uninfected persons lives in the same location as a subgroup of persons with active TB, for then any cases arising in the former subgroup would not be predicted by our model. This type of situation is likely to arise only in locations with very few active TB cases. Otherwise, the projection errors arise only from the extent to which the cross-spread of infection fails to cancel out (i.e., the net extent to which a subgroup infects others, over and above the number of the latter being infected by the former).

Although HIV status is addressed in this model, adding structure that more precisely reflects changes in transition rates of HIV-infected persons might also improve model projections. The model predicts that an increasing portion of cases will occur in Hispanics, and this prediction is supported by the most recent data, which show that the rate of decline in the number of cases in Hispanics is slower than for white non-Hispanics and for black non-Hispanics.

The foreign born account for a large fraction of the new cases seen in the United States (71). The model assumptions regarding the future contribution of the foreign born to the U.S. population are from the U.S. Bureau of the Census projections; therefore, this component of the population may be thought of as forming part of the backdrop against which the model projections are made. Departure from the patterns of immigration projected by the Census Bureau could cause two kinds of errors in model projections. A surge of immigration from countries with high rates of TB would soon be followed by increases in TB cases and case rates in the United States that would not be projected by the model. A different effect would be seen as a consequence of unexpected large increases in immigrants. Regardless of the TB status of the countries of origin, an increase in the number of immigrants would be reflected first in an artificial lowering of case rates because of the increase in denominators, followed by a longer-term slow increase in numbers of cases. The model could not recognize these changes within its current structure. These potential errors may be avoided as additional structure better reflecting immigrant status becomes incorporated into the model so that unexpected influxes may be modeled as exogenous inputs.

### Acknowledgment

The authors thank Philip J. Smith for helpful comments.

This work was supported in part by a contract with the Centers for Disease Control and Prevention under its cooperative agreement with the Association of Teachers of Preventive Medicine.

Dr. Debanne is an associate professor of epidemiology and biostatistics at the School of Medicine, Case Western Reserve University. She specializes in the construction of mathematical models of infectious diseases and has worked extensively in the area of tuberculosis.

### References

1. Bloch AB, Rieder HL, Kelly GD, Cauthen GM, Hayden CH, Snider DE Jr. The epidemiology of tuberculosis in the United States. Clin Chest Med 1989;10:297-313.
2. Ellner JJ, Hinman AR, Dooley SW, Fischl MA, Sepkowitz KA, Goldberger MJ, et al. Tuberculosis symposium: emerging problems and promise. J Infect Dis 1993;168:537-51.
3. Cantwell MF, Snider DE Jr, Cauthen GM, Onorato IM. Epidemiology of tuberculosis in the United States, 1985 through 1992. JAMA 1994;272:535-9.
4. Comstock GW. Variability of tuberculosis trends in a time of resurgence. Clin Infect Dis 1994;19:1015-22.
5. Centers for Disease Control and Prevention. Tuberculosis in the United States, 1997. Atlanta: July 1998:10-11.
6. Census of Population and Housing, 1990: Public use microdata sample U.S. technical documentation and machine-readable data file/prepared by the Bureau of the Census. Washington: The Bureau; 1992.
7. 1990 Census of Population and Housing, 5% and 1% public use microdata samples-U.S. geographic equivalency files machine-readable data files / prepared by the Bureau of the Census. Washington: The Bureau; 1994.
8. Census of Population and Housing, 1980; summary tape file 3A machine-readable data file / prepared by the Bureau of the Census. Washington: The Bureau; 1982.
9. Census of Population and Housing, 1980; summary tape file 3B machine-readable data file / prepared by the Bureau of the Census. Washington: The Bureau; 1982.
10. Census of Population and Housing, 1980; summary tape file 3C machine-readable data file / prepared by the Bureau of the Census. Washington: The Bureau; 1982.
11. Census of Population and Housing, 1980; summary tape file 3 technical documentation / prepared by the Data User Services Division, Bureau of the Census. Washington: The Bureau; 1982.
12. Census of Population and Housing, 1990: summary tape file 3 on CD-ROM machine-readable data files / prepared by the Bureau of the Census. Washington: The Bureau; 1992.
13. Intercensal estimates of the population of counties by age, sex, and race: 1980-1990 machine-readable data files / prepared by the Bureau of the Census. Washington: The Bureau; 1994.
14. Population projections for states, by age, sex, race, and hispanic origin: 1993 to 2020 machine-readable data file / prepared by the Bureau of the Census Population Division. Washington: The Bureau; 1994.
15. U.S. Bureau of the Census. Current population reports, P25-1111, population projections for states by age, sex, race, and hispanic origin: 1993 to 2020, by Paul R. Campbell. Washington: Government Printing Office; 1994.
16. Tuberculosis in the United States, for years 1980 through 1997, Atlanta (GA): Centers for Disease Control and Prevention, Division of Tuberculosis Control, 1982-1997.
17. AIDS Public Information Data Set, Statistics and Data Management Branch, Division of HIV/AIDS, CDC, 1993, Atlanta, GA.
18. Reports of Verified Cases of Tuberculosis (RVCT), CDC, Surveillance System for TB (SURVS-TB), Division of Tuberculosis Control, 1980-1993, Atlanta, GA.
19. Daniel TM, Debanne SM. Estimation of the annual risk of tuberculous infection for white men in the United States. J Infect Dis 1997;175:1535-7.
20. Markov AA. Investigation of a noteworthy case of dependent trials. Izv Akad Nauk Ser Biol 1907;1.
21. Markov AA. The calculus of probabilities. 4th ed. Moscow: GIZ; 1924.
22. Takács LF. Stochastic processes. London: Methuen, 1960.
23. Liaw K-L, Frey WH. Destination choices of the 1985-90 young adult immigrants to the United States: importance of race, educational attainment and labor market forces. International Journal of Population Geography 1998;4:49-61.
24. Muench H. Derivation of rates from summation data by the catalytique curve. Journal of the American Statistical Association 1934;29:25-38.
25. Styblo K. The relationship between the risk of tuberculous infection and the risk of developing infectious tuberculosis. Bulletin of the International Union against Tuberculosis 1985;60:117-9.
26. Styblo K. Epidemiology of tuberculosis. Selected papers. Vol 24. The Hague (The Netherlands): Royal Netherlands Tuberculosis Association (KNCV); 1991. p. 49-50.
27. Tuberculosis Program Management in the United States, 1984. Atlanta (GA): Centers for Disease Control and Prevention, Center for Prevention Services, Division of Tuberculosis Control; 1986.
28. Edwards LB, Acquaviva FA, Livesay VT, Cross FW, Palmer CE. An atlas of sensitivity to tuberculin, PPD-B, and histoplasmin in the United States. Am Rev Respir Dis 1969;99 Suppl:1-132.
29. Edwards LB, Smith DT. Community-wide tuberculin testing in Pamlico County, North Carolina. Am Rev Respir Dis 1965;92:43-54.
30. Rosenthal SR, Afremow ML, Nikurs L, Loewinsohn E, Leppmann M, Katele E, et al. BCG vaccination and tuberculosis in students of nursing. Am J Nurs 1963;63:88-93.
31. Hanzel GD, Rogers KD. Multiple-puncture and Mantoux tuberculin tests in high school students. A comparative study. JAMA 1964;190:1038-42.
32. Rhoades ER, Alexander CP. Reactions to the tuberculin time test in air force recruits. An analysis of 193,856 tests. Am Rev Respir Dis 1968;98:837-41.

33. Comstock GW. Frost revisited: the modern epidemiology of tuberculosis. Am J Epidemiol 1975;101:363-82.
34. Reichman LB, O'Day R. Tuberculous infection in a large urban population. Am Rev Respir Dis 1978;117:705-12.
35. Engel A, Roberts J. Tuberculin skin test reaction among adults 25-74 years, United States, 1971-1972. DHEW Publication No. (HRA) 77-1649.
36. Comstock GW, Cauthen GM. In: Reichman LB, Hershfield ES, editors. Tuberculosis: a comprehensive international approach. New York: Marcel Dekker, Inc.; 1993. p. 23-48.
37. Zeidberg LD, Gass RS, Dillon A, Hutcheson RH. The Williamson County tuberculosis study. A twenty-four-year epidemiologic study. Am Rev Respir Dis 1963;87 Suppl:1-88.
38. Ferebee SH. Controlled chemoprophylaxis trials in tuberculosis. A general review. Advances in Tuberculosis Research 1970;17:28-106.
39. Devadatta S, Dawson JJY, Fox W, Janardhanam B, Radhakrishna S, Ramakrishna CVL, et al. Attack rate of tuberculosis in a 5-year period among close family contacts of tuberculous patients under domiciliary treatment with isoniazid plus PAS or isoniazid alone. Bull World Health Organ 1970;42:337-51.
40. Morris SI. Tuberculosis as an occupational hazard during medical training: report of case-finding and follow-up study, with effective control program for tuberculosis in women medical students. American Review of Tuberculosis 1946;54:140-58.
41. Myers JA. Tuberculosis. A half century of study and conquest. St. Louis (MO): Warren H. Green, Inc.; 1970. p. 166-99.
42. Ferebee SH, Mount FW. Tuberculosis morbidity in a controlled trial of the prophylactic use of isoniazid among household contacts. Am Rev Respir Dis 1962;85:490-521.
43. Daley CL, Small PM, Schecter GF, Schoolnik GK, McAdam RA, Jacobs WR Jr, et al. An outbreak of tuberculosis with accelerated progression among persons infected with the human immunodeficiency virus. N Engl J Med 1992;326:231-5.
44. Di Perri G, Cruciani M, Danzi MD, Luzatti R, De Checchi G, Malena M, et al. Nosocomial epidemic of active tuberculosis among HIV-infected patients. Lancet 1989;2:1502-4.
45. Pitchenik AE, Burr J, Laufer M, Miller G, Cacciatore R, Bigler WJ, et al. Outbreaks of drug-resistant tuberculosis in an AIDS center. Lancet 1990;336:440-1.
46. Bielefeld RA. Fuzzy representation of uncertainty in disease progression [Ph.D. dissertation]. Department of Epidemiology and Biostatistics. Cleveland (OH): Case Western Reserve University; 1992.
47. Bielefeld RA. Estimation of HIV infection and AIDS onset times using fuzzy arithmetic techniques. In: Proceedings of the Seventh World Congress on Medical Informatics. Amsterdam (North-Holland); 1992. p. 921-7.
48. Bielefeld RA, Debanne SM, Rowland DY. Storing sparse and repeated data in multivariate Markovian models of tuberculosis spread. Computers and Biomedical Research 1996;29:85-92.
49. Adel'son-Vel'skii GM, Landis EM. An algorithm for the organization of information. Dokl Acad Nauk SSSR Math 1962;146:263-6.
50. Standish TA. Data structure techniques. Reading (MA): Addison-Wesley; 1980. p. 108-18.
51. Waaler H, Geser A, Anderson S. The use of mathematical models in the study of the epidemiology of tuberculosis. Am J Public Health 1962;52:1002-13.
52. Waaler HT, Piot MA. The use of an epidemiologic model for estimating the effectiveness of tuberculosis control measures: sensitivity of the effectiveness of tuberculosis control measures to the coverage of the population. Bull World Health Organ 1969;41:75-93.
53. ReVelle CS, Lynn WR, Feldman F. Mathematical models for the economic allocation of tuberculosis control activities in developing countries. Am Rev Respir Dis 1967;96:893-909.
54. Azuma Y. A simple simulation model of tuberculosis epidemiology for use without large-scale computers. Bull World Health Organ 1975;52:313-22.
55. Styblo K. The global aspects of tuberculosis and HIV infection. Bulletin of the International Union against Tuberculosis and Lung Disease 1990;65:28-32.
56. Schulzer M, Fitzgerald JM, Enarson DA, Grzybowski S. An estimate of the future size of the tuberculosis problem in sub-Saharan Africa resulting from HIV infection. Tuber Lung Dis 1992;73:52-8.
57. Heymann SJ. Modeling the efficacy of prophylactic and curative therapies for preventing the spread of tuberculosis in Africa. Trans R Soc Trop Med Hyg 1993;87:406-11.
58. Murray CJL, Styblo K, Rouillon A. Tuberculosis in developing countries: burden, intervention and cost. Bull Internat Union against Tuberc 1990;65:2-20.
59. Blower SM, McLean AR, Porco TC, Small PM, Hopewell PC, Sanchez MA, Moss AR. The intrinsic transmission dynamics of tuberculosis epidemics. Nature Medicine 1995;1:815-21.
60. Blower SM, Small PM, Hopewell PC. Control strategies for tuberculosis epidemics: New models for old problems. Science 1996;273:497-500.
61. Murray CJL, Salomon JA. Expanding the WHO tuberculosis control strategy: rethinking the role of active case-finding. International Journal of Tuberculosis and Lung Disease 1998;2:S9-15.
62. Murray CJL, Salomon JA. Modeling the impact of global tuberculosis control strategies. Proc Natl Acad Sci U S A 1998;95:13881-6.
63. Brewer TF, Heymann SJ, Colditz GA, Wilson ME, Auerbach K, Kane D, Fineberg HV. Evaluation of tuberculosis control policies using computer simulation. JAMA 1996;276:1898-903.
64. Frost WH. The age selection of mortality from tuberculosis in successive decades. American Journal of Hygiene 1939;30:91-6.
65. Ferebee SH. An epidemiological model of tuberculosis in the United States. Bulletin of the National Tuberculosis Association 1967 (January):4-7.
66. Rieder HL. Methodological issues in the estimation of the tuberculosis problem from tuberculin surveys. Tuber Lung Dis 1995;76:114-21.

67. Gammaitoni L, Nucci MC. Using a mathematical model to evaluate the efficacy of TB control measures. Emerg Infect Dis 1997;3:335-42.
68. MacIntyre CR, Plant AJ. Preventability of incident cases of tuberculosis in recently exposed contacts. International Journal of Tuberculosis and Lung Disease 1998;2:56-61.
69. Salpeter EE, Salpeter SR. Mathematical model for the epidemiology of tuberculosis, with estimates of the reproductive number and infection-delay function. Am J Epidemiol 1998;142:398-406.
70. West RW, Thompson JR. Modeling the impact of HIV on the spread of tuberculosis in the United States. Math Biosci 1997;143:35-60.
71. Liu Z, Shilkret KL, Tranotti J, Freund CG, Finelli L. Distinct trends in tuberculosis morbidity among foreign-born and U.S.-born persons in New Jersey, 1986 through 1995. Am J Public Health 1998;88:1064-7.
